# Impact of naturally spawning captive-bred Atlantic salmon on wild populations: depressed recruitment and increased risk of climate-mediated extinction

**DOI:** 10.1098/rspb.2009.0799

**Published:** 2009-07-29

**Authors:** Philip McGinnity, Eleanor Jennings, Elvira deEyto, Norman Allott, Patrick Samuelsson, Gerard Rogan, Ken Whelan, Tom Cross

**Affiliations:** 1 Department of Zoology, Ecology and Plant Science, University College Cork, Ireland; 2 Aquaculture and Catchment Management Services, Marine Institute, Newport, County Mayo, Ireland; 3 Centre for the Environment, School of Natural Science, Trinity College, Dublin 2, Ireland; 4 Department of Applied Sciences, Dundalk Institute of Technology, Dundalk, Ireland; 5 Rossby Centre, Swedish Meteorological and Hydrological Institute, 60176 Norrköping, Sweden

**Keywords:** captive breeding, Atlantic salmon, climate change, bioenergetics

## Abstract

The assessment report of the 4th International Panel on Climate Change confirms that global warming is strongly affecting biological systems and that 20–30% of species risk extinction from projected future increases in temperature. It is essential that any measures taken to conserve individual species and their constituent populations against climate-mediated declines are appropriate. The release of captive bred animals to augment wild populations is a widespread management strategy for many species but has proven controversial. Using a regression model based on a 37-year study of wild and sea ranched Atlantic salmon (*Salmo salar*) spawning together in the wild, we show that the escape of captive bred animals into the wild can substantially depress recruitment and more specifically disrupt the capacity of natural populations to adapt to higher winter water temperatures associated with climate variability. We speculate the mechanisms underlying this seasonal response and suggest that an explanation based on bio-energetic processes with physiological responses synchronized by photoperiod is plausible. Furthermore, we predict, by running the model forward using projected future climate scenarios, that these cultured fish substantially increase the risk of extinction for the studied population within 20 generations. In contrast, we show that positive outcomes to climate change are possible if captive bred animals are prevented from breeding in the wild. Rather than imposing an additional genetic load on wild populations by releasing maladapted captive bred animals, we propose that conservation efforts should focus on optimizing conditions for adaptation to occur by reducing exploitation and protecting critical habitats. Our findings are likely to hold true for most poikilothermic species where captive breeding programmes are used in population management.

## 1. Introduction

The natural environment has been undergoing unprecedented change due to climate warming (Fischlin *et al*. 2007). A large body of evidence indicates that many organisms, including fish, are affected by changes associated with this warming (Walther *et al*. 2002). Impacts include phenological change, biogeographical range shifts, reduced fitness and population extinctions (Beaugrand *et al*. 2003; Parmesan & Yohe 2003; Thomas *et al*. 2004; Perry *et al*. 2005). The focus of much contemporary research has been on adaptive responses to climate warming such as thermal tolerance (Portner & Knust 2007), acclimation to higher summer temperatures (Stillman 2003) and climate-mediated susceptibility to disease (Hari *et al*. 2006; Pounds *et al*. 2006). It has also been shown that adaptive shifts in the timing of life-stages, such as dormancy, migration, development and reproduction, will be required if animals are to adapt to projected future climates and that these life-stage adaptations should precede other effects (Bradshaw & Holzapel 2006).

Populations undergoing adaptation to new environmental conditions will incur demographic costs (Burger & Lynch 1995). Recruitment declines that follow will usually, with other factors such as over-harvesting or habitat deterioration, elicit a directed management response. For example, in the case of salmonids, every year millions of hatchery fish are deliberately released into the wild from captive breeding programmes (Myers 2004), either for the maintenance of fisheries and attempted enhancement of declining wild stocks, or also accidentally as a by-product of salmon farming (Schiermeier 2003). It may be that some of these management actions do more harm than good.

It has previously been shown that the progeny of farm-bred salmon are less fit than wild fish under natural conditions and that fitness reduction and potential extinction for wild populations could result from interactions with escaped farm salmon (McGinnity *et al*. 2003; Araki *et al*. 2007), although the mechanisms causing the reductions of fitness observed were not identified. Here we report on a 37-year study of Atlantic salmon (*Salmo salar*) from the Burrishoole river system in the west of Ireland, which shows that a wild population, where fish from a captive-bred population represent a variable but often substantial proportion of the naturally spawning cohort, is less able to adapt to higher freshwater winter temperatures and is negatively and disproportionately impacted by climate change, to such an extent that under projected future climates its future existence is threatened.

## 2. Material and methods

### (a) Study site

**Figure 1.**
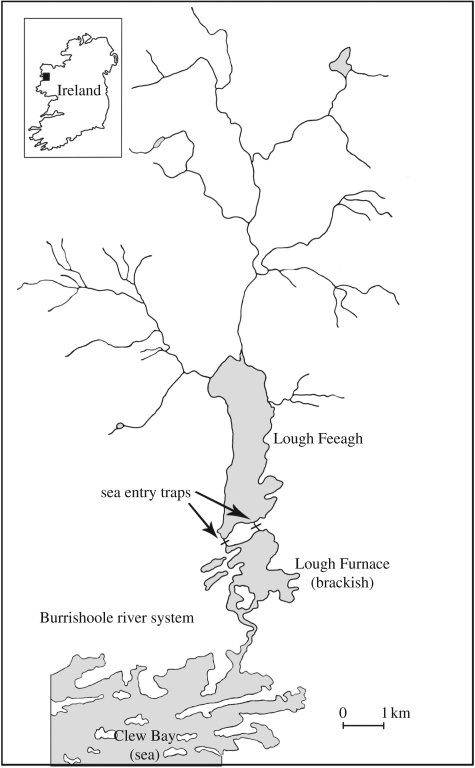
Map of the Burrishoole river system, County Mayo, Ireland, map showing the location of Lough Feeagh, Lough Furnace and the sea entry salmon traps.

The Burrishoole river system is situated in NW Ireland (53°59′ N, 09°37′ W) (figure 1). Lough Feeagh, the largest lake in the catchment, has a surface area of 3.9 km^2^, a mean depth of 14.5 m and drains an area of 83 km^2^. Burrishoole is situated close to the Atlantic coast and has a temperate oceanic climate. Average annual rainfall at the meteorological station on the eastern shore of Lough Furnace (figure 1) is 1560 mm year^−1^ while mean summer and winter water temperatures are 15°C and 6°C, respectively.

### (b) Salmon data

**Figure 2.**
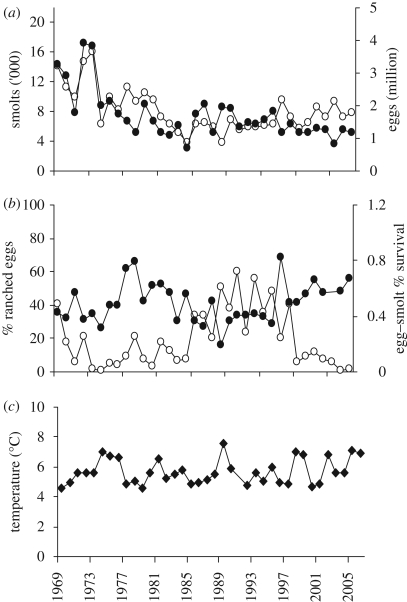
Smolt and egg numbers and winter temperatures at Burrishoole since 1972. (*a*) smolt numbers ('000, open circle) and egg numbers (million, filled circle) for that smolt cohort plotted against smolt migration year, 1972–2006; (*b*) egg to smolt per cent survival (filled circle) and the percentage of ranched eggs (open circle) in that egg cohort plotted against smolt migration year, 1972–2006; (*c*) mean winter (DJF) SWT (°C) in Lough Feeagh, 1972–2006.

Adult Atlantic salmon in the Burrishoole river system are anadromous and typically return to freshwater between June and September and spawn the following December. At this latitude (53°59′ N), juveniles generally spend three winters in freshwater, including the winter when eggs are in the gravel, before migrating to sea as smolts in April and May (Metcalfe & Thorpe 1990). A total trapping system for the counting of returning adults and migrating smolts has been operated at Burrishoole since 1969 at two locations above the tide (figure 1), allowing both the quantification of all fish entering and leaving the system and the estimation of egg numbers (figure 2*a*). The number of adult wild salmon spawning in the system during the period of the study ranged from 203 to 1485 individuals. Total estimates of egg input into the wild includes a variable proportion, 1–60%, of eggs deposited by adult salmon from an experimental captive breeding and smolt release (ranching) programme (figure 2*b*). The number of salmon bred in captivity spawning naturally in the system has varied between 8 and 608 fish. The captive breeding programme for salmon was established from wild fish collected from the catchment between 1960 and 1964. Additional wild fish were included in the breeding stock between 1970 and 1975. The breeding population has been effectively closed since that time with brood fish being selected from returning ranched fish. The Burrishoole hatchery population fish are referred to as ranched fish.

The estimated fecundities for this analysis of both Burrishoole wild and ranched females are based on values previously reported in 1974 of 4000 eggs for one sea winter fish and 6000 eggs for two sea winter fish (Salmon Research Trust/Agency of Ireland including the Marine Institute 1955–2006). The sex ratio of 55 per cent female for one sea winter wild fish is based on the average value reported between 1963 and 1974. A value of 70 per cent female is calculated for two sea winter wild fish based on observations on fish captured in the Burrishoole river traps. All ranched fish spawning in the wild are assumed to be one sea winter fish. All smolts are assumed to be 2 years old (time from emergence to smoltification). The percentage survival to smolt stage was calculated from these data and is referred to as egg-to-smolt survival. The value for 2001 was exceptionally high (3.5 times the s.d.) and was considered to be an outlier and omitted from the analysis. The number of ranched eggs contributing to the total number of eggs in any year was expressed as a proportion (ranched eggs/all eggs).

### (c) Water temperature and precipitation data

Surface water temperature (SWT) data for Lough Feeagh were collected from 1960 to present using a Negretti chart recorder and temperature probe from 1960 to 2003 at a site close to the outflow from the lake (depth 40–60 cm). Temperatures at 12.00 have been abstracted from charts since 1960. SWT has been measured at the same site using a Stowaway TidbiT (Onset Computer Corporation) from 2003 to present. Data were missing for several short periods including spring 1971 and January 1990. Monthly and seasonal means were calculated for 1970–2006. Daily precipitation data were available from the furnace meteorological station from 1960. Precipitation was summarized as monthly sums and as monthly 90th percentile values. The latter were considered to reflect high flow conditions.

### (d) Data analyses

Statistical analysis was performed using Minitab (13.1) or SPSS (11.0). We used a multiple linear regression approach to assess the influence of ranched eggs, water temperature and precipitation on egg-to-smolt survival, with egg-to-smolt survival as the dependent variable. Our initial exploration of the data indicated a significant negative relationship between egg-to-smolt survival and ranched egg proportion at the time of egg deposition. This factor was retained in subsequent models. Climatic variables were added in a forward running stepwise manner starting at the time of egg incubation (year 0) and continuing until the time of smolt migration to sea (June year 2). These were assessed as both monthly and seasonal values. The resulting model is referred to as model A. Two-way interactive terms were also assessed in the final model but none were significant.

In a further exploration of the data, we expressed the variable ranched eggs/all eggs as a dummy variable 1 or 0 for years with high and low proportions of ranched eggs (model B). A cut-off of 15 per cent gave two datasets with average proportion of 34 per cent (range 18–60%; *n* = 15) and 6 per cent (range 1–13%; *n* = 16) for high and low years, respectively, and was selected following visual inspection of the plot of ranched eggs/all eggs against egg-to-smolt survival. The dummy variable is referred to as HiLo and was retained in all further models. Climatic variables were then assessed in a stepwise manner as described above. This approach gave a model (model B) with results similar to those of model A, but explained a lower proportion of the variability. However, the final model included a significant interactive term between HiLo and water temperature at the time of egg incubation. The resulting model may be considered to represent two linear models for high and low ranched egg proportions, respectively (see below). Non-significant contributing terms are included in the model in accordance with the hierarchical principle. All datasets were assessed for normality using the Shapiro–Wilks test. All had a normal distribution, with the exception of reared eggs/all eggs. However, as the use of transformed data did not change the overall regression results, the original data were retained.

The models were as follows:

Low contribution of ranched eggs

Egg to smolt % survival = (−0.050 * Jan SWT year 0) + (−0.092 * Jan SWT year 1) + (0.131 * Spring SWT year 2) + (0.010 * 90 ppt Jan year 1) + (−0.011 * 90 ppt Aug year 1) + 0.265.

High contribution of ranched eggs

Egg-to-smolt survival = (−0.151 * Jan SWT year 0) + (−0.092 * Jan SWT year 1) + (0.131 * Spring SWT year 2) + (0.010 * 90 ppt Jan year 1) + (−0.011 * 90 ppt Aug year 1) + 0.606.

### (e) Future climate modelling

We used the lake temperature model DYRESM (Imberger & Patterson 1981; Imerito 2007), to model the impact of projected climate change on lake SWT. The model was run on a daily time step. Once validated, DYRESM was run using daily data for a control period and a future climate scenario period (2071–2100), a standardized control time frame commonly used in climate change impact studies across Europe (Räisänen *et al*. 2004). These daily data were multiple time-series output from the Climate Research Unit (UK) weather generator (Jones & Salmon 1995). The weather generator was first trained using local data for the period 1966–1993. Multiple 30-year time series of daily data were output for a control period based on these observational data. The weather generator was then perturbed based on the output from two general circulation model (GCM) and regional climate model (RCM) combinations to produce multiple 30-year time series for future climate scenarios. The RCM used for both combinations was the Rossby Center Regional Climate Model RCAO (Räisänen *et al*. 2004). The GCMs were the ECHAM4/OPYC3 GCM (Max Plank Institute, Germany) and the HadAM3H GCM (Hadley Center, UK). The future climate scenario period was run with assumptions based on the A2 and B2 scenarios on future greenhouse emissions. The training set consisted of 28 years from weather stations at Burrishoole and Belmullet, County Mayo (40 km NNW of Burrishoole). Daily mean lake SWT was simulated for the control and the projected climate period for all scenarios. Mean monthly and spring SWT were calculated from these data.

Future egg-to-smolt survival was then calculated using model B. Projected mean January and mean Spring lake SWT data from DYRESM runs, together with projected 90th percentiles of January and August precipitation, were used as input to model B to calculate projected egg-to-smolt survival for two reared egg scenarios based on high and low ranched egg contributions as defined above. The use of this regression gave two linear models for scenarios with less and greater than 15 per cent reared eggs, respectively.

Each 30-year period of daily data output by the weather generator gave 28 egg to smolt per cent survival estimates. The model was run for two future climate scenarios, which represent a relatively high and relatively low projected increase in water temperatures at Burrishoole, respectively: the ECHAM4/OPYC3-RCAO A2 scenario (E A2) and the HadAM3H-RCAO B2 scenario (H B2). Results are presented for 15 runs for the control period, the E A2 scenario and the H B2 scenario as box plots of the overall distribution of egg-to-smolt survival (*n* = 420).

## 3. Results

### (a) Factors impacting egg-to-smolt survival 1971–2006

The regression model results indicate that 76 per cent of the variability in egg-to-smolt survival in the Burrishoole salmon population for the period between 1969 and 2005 was explained by six factors (table 1, model A). These were five climate-related factors and the proportion of eggs in the natural spawning population contributed by ranched fish. The proportion of eggs from ranched fish in the spawning population had a significant negative impact on survival (*p* ≤ 0.0001). The five climatic effects included both positive and negative relationships. Both increases in winter water temperatures in year 0, when eggs are incubating in gravel beds, and in year 1, when fish are 0+ juveniles (parr), had significant negative effects on survival rates (*p* ≤ 0.0001). In contrast, higher water temperatures in the spring before the smolts went to sea had a positive impact on survival (*p* ≤ 0.0001). Two rainfall-related impacts were also identified. These were less significant than the temperature effects. Higher precipitation in the first winter had a positive effect on survival (*p* < 0.01) while a further negative effect on survival was related to increased precipitation in the August preceding the second winter (*p* < 0.0021).

**Table 1.** Impact of climatic variables and numbers of ranched fish on egg-to-smolt survival. Model A: regression of egg to smolt per cent survival on the proportion of eggs from ranched fish (ranched eggs/all), mean January SWT (°C) in year 0, mean January SWT in year 1, mean spring (MAM) SWT in year 2, the 90th percentile (90% ppt) of January precipitation (mm) in year 1 and the 90th percentile of August precipitation in year 1; model B: as A but with a dummy variable (HiLo) in place of the proportion of ranched eggs.

	coefficient	s.e.	*T*	*p*-value	d.f.	*R*^2^ adjusted
*model A*						
constant	0.616	0.147	4.20	0.0003	24	0.76
ranched eggs/all	−0.620	0.083	−7.52	≤0.0001		
SWT Jan year 0	−0.084	0.016	−5.77	≤0.0001		
SWT Jan year 1	−0.109	0.018	−5.97	≤0.0001		
SWT Sep year 2	0.121	0.019	6.28	≤0.0001		
90% ppt Jan year 1	0.013	0.005	2.84	0.0090		
90% ppt Aug year 1	−0.011	0.003	−3.44	0.0021		
*model B*						
constant	0.265	0.200	1.32	0.1982	23	0.68
HiLo	0.341	0.170	2.01	0.0563		
SWT Jan year 0	−0.050	0.023	−2.20	0.0380		
SWT Jan year 1	−0.092	0.021	−4.49	0.0002		
SWT Sep year 2	0.131	0.023	5.69	≤0.0001		
90% ppt Jan year 1	0.010	0.005	1.9	0.0703		
90% ppt Aug year 1	−0.011	0.004	−2.82	0.0097		
HiLo × SWT J year 0	−0.101	0.032	−3.13	0.0047		

A further exploration of the data using an indicator variable (‘HiLo’) distinguished between the years with high (mean = 34%) and low (mean = 6%) proportions of ranched fish in the breeding population (table 1, model B). This model explained less variability in egg-to-smolt survival than model A (*R*^2^ = 0.68 compared with *R*^2^ = 0.76), but showed a significant interaction effect (*p* < 0.005) between HiLo and water temperature in the first winter (*p* < 0.005).

### (b) Projected future climate at Burrishoole

**Figure 3.**
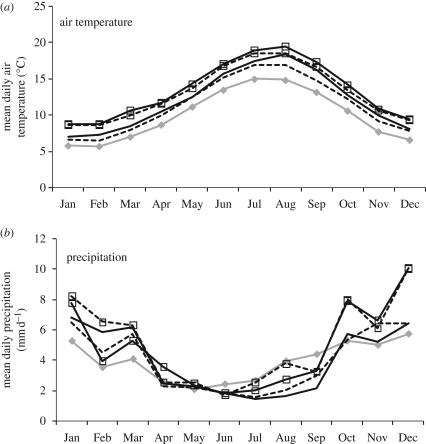
(*a*) Mean daily air temperature and (*b*) precipitation at Burrishoole for a control period (1968–1997, grey line with filled diamond) and for a future climate period (2071–2100) for four GCM–RCM scenario combinations namely the E A2 (ECHAM 4 GCM and A2 SRES emission scenarios) and H B2 (HadA3 GCM and B2 SRES emission scenarios). Solid line, H A2; dashed line, H B2; solid line with square, E A2; dashed line with square, E B2.

Future changes in climate for Burrishoole for 2071–2100 include an increase in air temperature in all seasons and changes in the seasonal distribution of precipitation (figure 3). The projected increase in January air temperatures ranges from 0.9°C to 3.1°C while increases for spring are expected to be between 1.2°C and 3.3°C. Precipitation in January is projected to increase by between 25 per cent and 58 per cent while in contrast August should be drier with decreases ranging from 4 per cent to 62 per cent. Increases in water temperature of between 0.8°C and 2.3°C are projected for January, while those for spring (March, April and May) range from 0.8°C and 2.4°C.

### (c) Projected egg-to-smolt survival at Burrishoole

**Figure 4.**
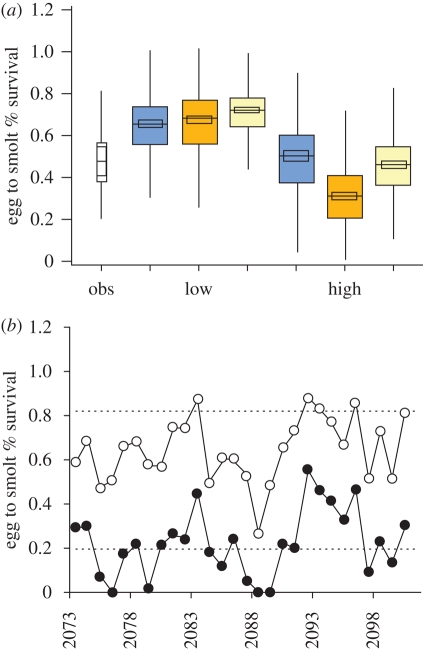
Projected impact of climate change on survival. (*a*) Modelled egg to smolt per cent survival based on regression model B (table 1) for low and high ranched egg proportions for a control period (blue) and two future climate scenarios (*n* = 420) (orange, E A2; yellow, H B2) and observed egg to smolt per cent survival for the period 1972–2006; inner area = 95 per cent CI for the median; (*b*) egg to smolt per cent survival for low (open circles) and high (closed circles) proportions of ranched eggs for one run based on the E A2 scenario. The area between the two broken lines is the range of observed values from 1972 to 2006.

Model B gave two linear models: one for a population with less 15 per cent eggs from reared fish and one for a population with greater than 15 per cent eggs from reared fish. Future egg-to-smolt survival estimated based on these models for two projected climate scenarios (E A2 and H B2) indicated no reduction in egg-to-smolt survival when the proportion of eggs from ranched fish was low under either scenario (figure 4*a*). Poor outcomes and considerably lower egg-to-smolt survival were predicted for the population when the proportion of eggs originating from ranched fish was high with 4 out of the 28 years (14.3%) having zero smolt recruitment from the eggs deposited (figure 4*b*).

## 4. Discussion

### (a) Adaptation in response to a changing and variable environment: a possible mechanism

In a recent review of local adaptation in Atlantic salmon, Garcia de Leániz *et al*. (2007) concluded that water temperature and photoperiod (and factors related to them) were likely to be the most important physical variables determining local selective pressures in Atlantic salmon populations. Our models show that higher water temperatures encountered in the first winter, when eggs are incubating in gravel beds, and in the second winter, when fish are 0+ juveniles (parr), have negative effects on survival. We believe that the negative response to warmer winter water temperatures found is consistent with Garcia de Leániz *et al*. (2007) and can be explained most effectively within a bioenergetics framework similar to the model proposed for the little brown bat *Myotis lucifugus* (Humphries *et al*. 2002), with the timing of the physiological processes associated with energy acquisition synchronized by photoperiod (Bradshaw & Holzapel 2006). That winter temperatures rather than summer temperatures were found in this study to have the greatest effect on survival supports the idea proposed by Bradshaw & Holzapel (2006) that climate change, particularly at higher latitudes, will impose seasonal rather than thermal selection (summer thermal tolerance) on natural populations.

As has also been reported recently for the European great tit *Parus major* (Nussey *et al*. 2005) warm winters can lead to earlier hatching but not necessarily advance the supply of food for the hatchlings. It is quite plausible that this is also the case for salmon eggs and salmon alevins, with earlier than normal hatching and emergence induced by increased winter water temperatures predisposing fry to the risk of starvation. It follows that higher mortality is likely to occur in warm winters because of a mismatch in developmental schedules between the hatching eggs and emerging salmon fry and their prey resulting in insufficient energy reserves for survival. The eggs of a ‘locally adapted’ population of salmon might be expected to match the energy demands of a typical winter by either having eggs of sufficient size (see below), by a slowing of developmental rates, or by a phenological adjustment of spawning time.

The interaction term in model B suggests that increases in winter temperature are a much greater problem for the progeny of hatchery fish, particularly the eggs, than for the progeny of wild fish. We know from previous studies that Burrishoole ranched salmon produce significantly smaller eggs than Burrishoole wild salmon (McGinnity *et al*. 1997), which would pre-dispose them to higher mortalities in the wild during winters with excessive energy demands as they would have less intrinsic food resources than larger eggs. While it has been demonstrated elsewhere (Heath *et al*. 2003) that there is usually no fitness penalty for producing small eggs in benign hatchery environments, less fit offspring would emerge from small eggs than large eggs in sub-optimal natural environments (Einum *et al*. 2004). Other adaptive traits such as the timing of spawning (Heggberget 1988) and genetically determined differences in predator avoidance in young fish (Johnsson *et al*. 2001) are in addition likely to contribute to the large apparent differences in performance between the progeny of ranched parents compared with their wild conspecifics.

For older fish experiencing their first winter as parr, our models also suggest a strong negative effect of increasing winter water temperature on survival. By proposing a simple ‘bio-energetics’ explanation for this outcome, we might reasonably assume that increases in metabolic rates as a consequence of higher winter temperatures cause the depletion of parr energy reserves faster than in cold years and decrease the likelihood of survival until food supplies become available in spring. Excessive depletion of energy reserves in winter has recently been shown to be a major cause of mortality in juvenile fish (Finstad *et al*. 2004) and provides strong support for this idea.

However, in common with many animals, salmon parr undergo a winter dormancy or hibernation to overcome the demands of winter stress. There are two advantages to hibernating: conserving energy that would be used up in seeking food and maintaining station at high flows and secondly in reducing the risk of predation. It has been shown previously that salmon stop feeding in the autumn and find shelter within the stream bed even though food is plentiful (Metcalfe & Thorpe 1992), suggesting that this behaviour is adaptive. Furthermore, Metcalfe & Thorpe (1992) have shown that the anorexia associated with the onset of winter is a genetically determined response to photoperiod. To survive, salmon parr need to predict accurately when food will be available some six months in advance. Photoperiod provides one of the most important cues for events that are distant in time or space (Bradshaw & Holzapfel 2008). In contrast, temperature, food and other ecological conditions become more important closer to the actual event itself and are associated with phenotypically plastic, non-genetic, phenological responses to the environment. Incorrect physiological or behavioural responses to photoperiod cues, such as suspending feeding prematurely, could explain the poorer survival in Burrishoole salmon associated with increased winter water temperature observed in this study. In contrast to the eggs, no HiLo interaction effect was found in overwintering parr. Either the warmer winter temperature affects both the parr progeny of wild and hatchery fish equally or more likely that the progeny of hatchery fish die at the egg stage disproportionately and are no longer present in the population in sufficient numbers to have an effect.

In the final winter before spring metamorphosis from parr to smolt, appetite and growth of salmon are maintained through the winter, and parr are therefore less dependent on stored energy reserves than in the first two winters. Consequently, it is not surprising that we found no relationship between water temperature in the third winter and survival. However, the positive effect on survival of warm spring temperatures may come about through improved foraging efficiency and enhancement of the smoltification process. Our model would suggest that there is little difference in the response of hatchery and wild presmolts, but the progeny of hatchery fish are less likely to have survived the rigours of their first two winters to have much impact on overall survival at the smolt stage, their number being greatly reduced at egg and fry stages.

### (b) The possibility that adaptation to environmental change in the wild is constrained by gene flow from the hatchery

Our results clearly demonstrate that hatchery fish spawning in the wild can impact negatively on freshwater survival chances in natural populations. The study provides some clues as to one specific cause and effect. However, in addition to the winter temperature effects postulated, captively bred fish in other studies have shown increased metabolic rates and food conversion efficiency (Thodesen *et al*. 1999; Handeland *et al*. 2003), higher growth rates (Fleming & Einum 1997; McGinnity *et al*. 1997), to be more aggressive (Einum & Fleming 1997; Fleming & Einum 1997) and less risk averse to predators compared with wild fish (Einum & Fleming 1997; Fleming & Einum 1997). These are all characteristics that have proven to be maladaptive in the wild. Without tangible gene flow from the hatchery to the river, the differential response of the progeny of wild and hatchery fish to higher winter temperature could account for the variation in survival observed with respect to the proportion of hatchery fish in the spawning population. Yet this is of little consequence to the evolutionary trajectory of the wild population unless there is a direct genetic interaction by inter-breeding between wild and ranched adults either in the parental F1 or subsequent generations. A direct interaction would lead to a change in the genetic composition of the wild population by the addition or incorporation of a ‘genetic load’ carried over from the hatchery into the wild that might subsequently affect its productivity. This ‘load’ or degree of maladaptation to the existing natural environment occurs because hatchery populations (where food is available ad libitum) do not experience the continuous evolutionary adjustment possible in the wild, and therefore they do not have the benefit of a history of natural selection experienced by the wild population. Consequently, the mismatch between the hatchery fish and the natural environment will increase. In addition, domestication selection in the hatchery population, such as for reduced fear of predators, will exacerbate these differences over time. Illustrating this possibility empirically (McGinnity *et al*. 1997, 2003), it has been shown that hybrid progeny of local wild salmon and farm escape salmon parents have, when compared under natural conditions, intermediate fitness to their parents. This lower fitness in hybrids was found to be accompanied by a reduction in the productive potential of the hybridized population. It is known that ranched and wild salmon at Burrishoole interbreed in the wild (Thompson *et al*. 1998), gene flow primarily occurring by fertilization of eggs of ranched females by wild mature male parr. Although not usually to the same extent, it has also been shown in this and other studies that substantial levels of gene flow also occurs via captive bred males to wild females (Fleming *et al*. 2000; Weir *et al*. 2004). The extent of the gene flow between ranched males and wild females might be expected to be a function of the proportion of captive bred fish in the spawning population. We might conclude therefore that some of the HiLo effect on egg-to-smolt survival pertains to both the progeny of the hatchery fish and the F(n) hybrid progeny of wild and hatchery reared fish.

We interpret our results as being consistent with the predictions of general theoretical demographic-evolutionary models (Burger & Lynch 1995; Gomulkiewicz & Holt 1995; Kirkpatrick & Barton 1997). These models suggest that each time a population undergoes an evolutionary adjustment in response to variations in the environment through natural selection, a demographic penalty, such as a higher rate of mortality is incurred. In our study, the demographic penalty associated with increases in winter temperature or high summer precipitation is a reduction in egg-to-smolt survival. Though the relationship between natural selection and environmental change and the demographic response have been modelled extensively, there are very few empirical examples under natural conditions (Grant & Grant 2002). The end product of these adjustments should be a population that is better adapted to its local environment and consequently is increasing in size towards its carrying capacity (Kirkpatrick & Barton 1997). However, what is also suggested from our results is that the potential to adapt to environmental change can be thwarted by concurrent and recurrent genetic changes in the wild population following hybridization with captive bred animals, which are subject to domestication and possibly directional selection. Also they will not be not subject to natural selection in benign hatchery environments and as a consequence are less well adapted to life in the wild. This would counteract any positive adaptive response in the wild population to environmental change and increase the demographic costs imposed by natural selection.

### (c) The risk of extinction and projected future climate scenarios

**Figure 5.**
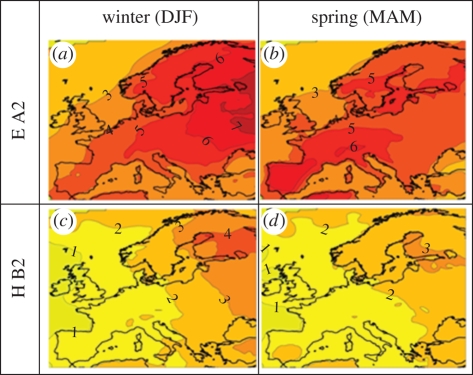
Projected change in air temperature for Europe: 2070–2100. The mean difference between scenario (2071–2100) and control (1961–1990) outputs for air temperature (°C) across Europe in winter (DJF) and spring (MAM) for the ECHAM4/OPYC3-RCAO A2 (E A2) (*a* and *b*) and HadAM3H-RCAO B2 (H B2) (*c* and *d*) scenarios.

Projected impacts of climate change for Western Europe include an increase in air temperature in all seasons (figure 5) and changes in the seasonal distribution of rainfall (Räisänen *et al*. 2004). Our simulations of projected changes in lake surface temperature based on these results indicate average increases of between 0.8°C and 2.3°C in January and 0.8°C and 2.4°C in spring (March, April and May). We used our model B to assess the egg-to-smolt survival prospects for the Burrishoole wild salmon population under two future climate scenarios, i.e. 60 years hence, with low and high proportions of Burrishoole ranched fish in the wild spawning cohort. The model outputs show no reduction in egg-to-smolt survival when the proportion of eggs from ranched fish is low under either of the future projected climate scenarios (figure 4*a*). If anything they suggest an increase in survival compared to historical levels. In contrast, poor outcomes and considerably lower egg-to-smolt survivals, including some catastrophic results are predicted for the population when the proportion of eggs originating from ranched fish is high. In 11 of the 15 30-year model runs based on the E A2 scenario, zero egg-to-smolt survival was observed in at least one year. Two of these model runs are even more pessimistic each showing two consecutive years with zero survival (figure 4*b*). The projected decreases in freshwater survival in combination with a continuation of recent declines in marine survival (Peyronnet *et al*. 2007) would intimate a population highly vulnerable to extinction at high levels of ranch fish input.

It has been shown that, in a directionally changing environment, mean phenotype will lag behind the optimum, but will evolve on a roughly parallel trajectory (Burger & Lynch 1995 and see reviews by Garcia de Leániz *et al*. 2007; Naish & Hard 2008). The magnitude of the lag determines the vulnerability to extinction. Based on our analysis, large scale interbreeding with captive-bred fish has the potential to increase the size of this disparity and prevent the population from at least tracking changes in the environment. Even low levels of gene flow from a captive population to a wild population will shift the wild population's mean phenotype so that it approaches the hatchery optimum (Ford 2002). The effects of this are particularly severe if high levels of hybridization and introgression are reoccurring on a continuous basis (Hindar *et al*. 2006). Furthermore, as the population adapts to a novel environment, its density may fall below critically low levels for a period of time (the burden imposed by natural selection). During this time, it becomes highly vulnerable to extinction by non genetic stochastic demographic processes, in other words ‘the population succumbs before it has a chance to evolve’ (Gomulkiewicz & Holt 1995). Our results suggest that high levels of gene flow from the hatchery can impair the adaptive capacity of the Burrishoole wild salmon population enough to jeopardize its long-term viability. The evidence that this occurs by impacting directly on traits associated with the fish's ability to deal with increased winter temperatures at the egg stage of development is compelling, though this is not to say that other important traits are not similarly affected by gene flow from the hatchery.

### (d) Management implications

The seemingly intuitive management response to declines in natural production for both salmon and other commercially exploited wild fish species has been to increase the production and release of ranched fish (Myers 2004) with the objective of enhancing or at least sustaining natural production (Bell *et al*. 2006). However, as we have shown here, such a practice is likely to exacerbate the impact of climate change on natural populations rather than ameliorate it (Araki *et al*. 2007). Furthermore, an estimated two million salmon escape from farms around the North Atlantic each year (Schiermeier 2003). It is surely a serious cause for concern that a large proportion of the marine production of farmed salmon occurs close to high-latitude Norwegian rivers. In these rivers, where salmon parr require four and even five winters to reach the smolt stage (Metcalfe & Thorpe 1990), climate projections show winter temperatures increasing by the greatest magnitude (up to 6°C) (figure 5). Our results imply that the impact of climate change combined with a high proportion of farm escapes that breed in the wild could have an even greater negative impact on the productivity of wild salmon populations at these high latitudes than predicted for western Ireland, particularly as each extra year spent in freshwater must increase the demographic cost of selection.

We conclude based on this study that ranched fish capable of breeding in the wild, even where strains have been established from local or progenitor stocks, should not be deliberately introduced into natural salmon rivers, and that measures must be found to reduce the numbers of escaped farm salmon in nature, if wild salmon populations are to successfully adapt to climate change. Our findings are likely to hold true for other poikilothermic animals where captive breeding programmes are used in population management. Recent reports suggest that captive breeding is being considered for some of the estimated 1900 species of amphibians that are known to be threatened with extinction (Frankham 2008; Stuart *et al*. 2008). It is pertinent here that much of the extinction risk for amphibians is likely to be also climate mediated (Pounds *et al*. 2006). Rather than imposing an additional genetic load on wild populations experiencing climate change by releasing captive bred animals, we propose that conservation efforts should focus on optimising conditions for adaptation to occur by reducing exploitation and protecting critical habitats.
